# The economic burden of maternal mortality on households: evidence from three sub-counties in rural western Kenya

**DOI:** 10.1186/1742-4755-12-S1-S3

**Published:** 2015-05-06

**Authors:** Aslihan Kes, Sheila Ogwang, Rohini Prabha Pande, Zayid Douglas, Robinson Karuga, Frank O Odhiambo, Kayla Laserson, Kathleen Schaffer

**Affiliations:** 1 International Center for Research on Women (ICRW), 1120 20th St NW, Suite 500 North, Washington, D.C. 20036, USA; 2 Kenya Medical Research Institute (KEMRI), Kisumu, Kenya; 3 Independent Consultant, 7118 Willow Avenue, Takoma Park MD 20912, USA; 4 LVCT Health, Tivoli Centre, P.O Box: 3294-40100, Kisumu, Kenya; 5 Global Disease Detection Program, Centers for Disease Control (CDC)*, 1600 Clifton Rd. Atlanta, GA 30329-4027, USA; 6 Family Care International, 588 Broadway #503, New York, NY 10012, USA

**Keywords:** Maternal death; Kenya; financial costs.

## Abstract

**Background:**

This study explores the consequences of a maternal death to households in rural Western Kenya focusing particularly on the immediate financial and economic impacts.

**Methods:**

Between September 2011 and March 2013 all households in the study area with a maternal death were surveyed. Data were collected on the demographic characteristics of the deceased woman; household socio-economic status; a history of the pregnancy and health care access and utilization; and disruption to household functioning due to the maternal death. These data were supplemented by in-depth and focus group discussions.

**Results:**

The health service utilization costs associated with maternal deaths were significantly higher, due to more frequent service utilization as well as due to the higher cost of each visit suggesting more involved treatments and interventions were sought with these women. The already high costs incurred by cases during pregnancy were further increased during delivery and postpartum mainly a result of higher facility-based fees and expenses. Households who experienced a maternal death spent about one-third of their annual per capita consumption expenditure on healthcare access and use as opposed to at most 12% among households who had a health pregnancy and delivery. Funeral costs were often higher than the healthcare costs and altogether forced households to dis-save, liquidate assets and borrow money. What is more, the surviving members of the households had significant redistribution of labor and responsibilities to make up for the lost contributions of the deceased women.

**Conclusion:**

Kenya is in the process of instituting free maternity services in all public facilities. Effectively implemented, this policy can lift a major economic burden experienced by a very large number of household who seek maternal health services which can be catastrophic in complicated cases that result in maternal death. There needs to be further emphasis on insurance schemes that can support households through catastrophic health spending.

## Introduction

In recent years, increasing attention has been directed to the social and economic impacts of poor maternal health, framing the issue as a broader development concern with impacts on women’s empowerment, household wellbeing, and economic and social development at a national level. To date, research has mostly focused on costs of maternal morbidity and obstetric care revealing substantive immediate and long term costs incurred by women and their households while only a few studies have explored the economic and financial costs of maternal mortality which can be particularly severe.

Often unexpected and likely to be accompanied by the addition of a newborn, maternal mortality is expected to set off a multitude of shocks to households’ economic wellbeing, particularly in rural economies where the household is the main economic unit providing most of its own subsistence needs and where there are seldom any social protection measures in place. In these settings, women have many economic roles; as producers and income earners, farmers and entrepreneurs, and carry out most reproductive (non-market household work) and care work [1]. The loss of women’s contributions combined with the spending shock they face can force households, particularly those already vulnerable, into poverty.

This paper presents findings from a two-year research study aimed to understand and measure the economic and social impacts of maternal mortality. The study was conducted in a poor rural setting in Western Kenya where maternal deaths continue to be alarmingly high. While decreased from 460 in 2004/2008, Kenya’s maternal mortality rate continues to be among the highest in Sub-Saharan Africa with 400 deaths per 100,000 live births in 2009/2013 [2]. This paper presents evidence in one of the key areas of impact; the financial costs associated with maternal morbidity and mortality and the economic consequences to households of these costs.

## Background

Research focusing on the social and economic costs of maternal mortality remains limited, in large part because of methodological constraints. One recent study in three provinces of China explored these costs and found that the direct costs of a maternal death were significantly higher than the costs of childbirth without a maternal death. Hospitalization and emergency care expenses represented the largest proportion of non-funeral direct costs [3]. Using macro estimates of individual contribution to national aggregate productivity, a four-country study in Africa estimated that productivity loss due to early maternal death ranged from USD $850 in Uganda to USD $1838 in Senegal [4].

A larger evidence-base exists on the impacts of adult female or parental death on households, particularly on children, and suggests that maternal mortality can impose significant immediate and long term costs. Studies in Bangladesh found that a mother’s death can have a greater negative impact on child survival than a father’s, with the children whose mother died being more likely to die than those who had fathers who died [5-7]. Studies in sub-Saharan Africa found that adult deaths, primarily due to an AIDS-related illness, had significant negative impacts on children’s survival, health and schooling [8,9], and that the effects are particularly strong for children who lost their mothers [10-12]. Children whose mothers had died were also more likely to not be enrolled in school, as they became substitutes for the deceased woman in her labor and productive activities [13]. Research in Tanzania found that maternal death impacted children’s nutrition (resulting from children not being breastfed), decreased education as it is often mothers who prioritized and supervised education in the household), decreased access to healthcare, and that girls, in particular, were more susceptible to risk of early marriage and high-risk sexual behavior [14].

The death of an adult male or female can also have adverse effects on the level of household consumption because of reduced resources for growing and purchasing food. As with consequences of adult death for children, the effects on household consumption and resources were found to be larger when the death is that of a woman [13,15,16]. A study in Malawi found that a maternal death from AIDS disrupts household members’ time allocation, and that other women in the household, in particular, need to increase the time they allocate to economic activities and household chores [17]. Naidu and Harris also found that reallocating household labor is a common strategy to cope with the loss of a productive adult [18]. In particular, elders may have to go back to work, and children may be pulled out of school to help. Basu [15] found in her Delhi-based study that it was often difficult for the household to mitigate the consequences of the death of an adult woman because men were not accustomed to managing the household and its budget. This kind of potentially disruptive reallocation may ease once households adjust to their new situation [19], but longer-term studies to establish this pattern are rare.

## Study site

The fieldwork took place between 2011-13 in Rarieda, Gem and Siaya sub-counties, lying northeast of Lake Victoria in Siaya County, Kenya. The area remains heavily reliant on subsistence farming and is characterized by high levels of poverty. Limited employment opportunities often lead to outmigration, particularly among younger populations contributing to the diffused household structure common in the region [20,21]. The area is also characterized by poor health outcomes among its population. The Nyanza region ranks among the highest in terms of HIV, tuberculosis and malaria rates in the country and has some of the worst indicators for child and overall health status. The region is also one of the most poorly served provinces in terms of public health facilities [22,23].

Table 1 shows select comparative demographics between Kenya as a whole and Nyanza province, from the Kenya DHS conducted in 2008-09.

**Table 1:** Kenya and Nyanza Province, 2008-09

	Nyanza	Kenya
**Childbearing**		
*% of 15-19 yr. olds who have started childbearing*	27	17.7
*Median age at first sexual intercourse* (*women age 25-49 yrs.*)	16.5	18.2
*Median age at first birth* (*women age 25-49 yrs.*)	19	19.8

**Maternal care**		
*% who received antenatal care from a skilled provider^1^*	93.6	91.5
*% delivered in a health care facility* (*public or private*)*^2^*	44.2	42.6
*% who had no postnatal checkup^1^*	65.8	52.6

**Fertility and family planning**		

*Total fertility rate*	5.4	4.6
*% of women using any modern contraception*	32.9	39.4

Source: Kenya National Bureau of Statistics (KNBS) and ICF Macro. 2010. *Kenya Demographic and Health Survey* 2008-09.

1. % of women 15-49 with a live birth in the 5 years preceding the survey

2. % distribution of live births in 5 years preceding the survey

Desai et al, in their analysis on the causes of death among women of childbearing age in the Nyanza province, found that about one-third of these were due to direct obstetric causes producing a mortality ratio of 740 per 100,000 live births over six years [24]. van Eijk el al. [20] found that maternal healthcare utilization among women in Nyanza is considerably higher during the antenatal period compared to delivery and postpartum. In their study in Gem and Asembo, the authors found that 90% of 571 women visited an Antenatal Clinic at least once during their pregnancy, with a median number of 4 visits. Yet, 83% delivered outside of a health facility, a pattern common nationwide.

## Data and methods

### Study sample

The study sample was selected from KEMRI/CDC’s Health and Demographic Surveillance System (HDSS), established in 2001. The HDSS currently includes a total population of 225,000 individuals in Rarieda, Gem and Siaya sub-counties who are visited quarterly. In 2008, 41.7 of the population was between the ages of 15-49 years, of which slightly over half (54) were women of reproductive age [24].

HDSS uses Global Positioning System (GPS) coordinates to map each compound in the surveillance area and assign each household and individual within these compounds a unique identification number. Information collected through the quarterly survey includes data on births, deaths and the causes of death (through verbal autopsy), pregnancy, pregnancy outcomes, morbidity, migration, education and socioeconomic status.

The sampling frame inherent in this, as in other surveillance data removes many of the biases found in study designs that are not based on a whole population sample. Correct identification of maternal deaths is assured as the HDSS uses the WHO Verbal Autopsy method, and collects data on cause of death through an experienced team of community interviewers, village reporters and staff responsible for quality control.

Since maternal death is a rare event, the study attempted to identify and interview respondents about all maternal deaths that occurred within the surveillance area in a period of 22 months, the duration of the data collection period of the study. To minimize recall issues, households were recruited on a rolling basis, after a period of at least 2 months after the maternal death to ensure they were respected during time of grief but as soon as possible after that death. Specifically, households were approached no sooner than two months after the maternal death, but no later than six months.

Two control households were interviewed per each case household. Control households were defined as those where a woman had a healthy pregnancy and delivery in the same time period as the women from the case households.

Due to ethical considerations around patient identification and findings of a preliminary scan of select health care facilities in the study areas that pointed at significant lack of documentation, the study chose not collect provider level data. Information on the medical course of the pregnancy, delivery and postpartum periods was collected at the household level and lacked clinical detail.

### Data collection

Two survey questionnaires were used to collect data from all identified case and control households. *The cost questionnaire* collected detailed information on the types of care sought during pregnancy, childbirth and postpartum, and the costs associated with each incidence of help seeking, i.e. health care utilization. It was designed to capture expenditures associated with using a range of services; institutional services; hospitals, health centers and private clinics as well as services by non-institutional providers such as traditional birth attendants (TBAs) and informal medical practitioners. Expenditures associated with home-based delivery were also surveyed.

For each incidence of care sought, cost information was collected on a detailed list of items, including spending on transportation and other medical and non/medical expenses related to the visit (see Table 2). In households that experienced maternal mortality, an additional module was administered to collect information about funeral costs. While most cost data were collected in monetary terms, where in-kind payments are common, such as payment to informal sector providers and funeral expenses, households were asked about the monetary equivalent of the spending incurred.

**Table 2:** Classification of Costs

Types of Costs	Examples
Facility/service based	Admission fees; File/card fees; Informal fees; Ambulance (for referred patients); Other medical costs at the health center

Informal service providers	Aggregate total payment to person/people who provided the service

Transport costs (for both the women and companions, if any)	

Other medical/non-medical costs (for both the women and companions, if any)	Medicines for women purchased outside a facility; food; bedding; hotel stay; other purchases

Funeral costs	Costs incurred in holding the funeral service such as food (for guests), rental of chairs, etc.

*The SES questionnaire* collected socio-economic information on women and their household including household expenditure on food and non-food items and durable goods, household asset ownership and dwelling characteristics. Case households were also asked about the members’ employment and time use.

Interviews were conducted with an adult household member aged 18 years or older who was most knowledgeable about the information sought. If one person was not knowledgeable in all topic areas, the questionnaires allowed for different respondents for different modules. Finally, even though enumerators were instructed to ask for receipts, most of the time, information collected relied on respondents’ memory. An important caveat to note is that while women in control groups report on their own experiences, the most knowledgeable people in the case households reported on the experiences of the deceased and the aftermath of her death.

During the 22-month data collection period, 67 households that had a maternal death were identified using the sampling strategy described above. The sample size of the control households was 92, matched by timing of birth such that the control household for a maternal death household includes a woman who delivered in a period of up to two months after the maternal death. Of the identified, 59 cases and 86 controls were interviewed using the tools. The response rate for the SES questionnaire was slightly lower among case households with a total of 54 interviews fully or partially completed. The remaining households refused to be interviewed or had no appropriate respondent to take the survey

Group discussions were also held with 11 of the case households. An attempt was made to select households to include a mix of socio-economic strata and households representing a variety in regard to characteristics such as number, age and sex of children. No group discussions were held with control households. These households were interviewed using an open-ended interview guide to collect information on the living and working arrangements of household members before and after the maternal death.

### Methodology

The methodology integrated several approaches to respond to the specific features of the study context; a rural setting in a developing country with potential low service utilization, lagging administrative record-keeping in facilities, and where labor markets are highly informal. Overall, a similar methodological approach to Ye et al [3] was followed, particularly in the measurement of the direct costs of maternal costs.

Specifically, similar to Ye et al, direct costs were measured in terms of the out-of-pocket expenditures related accessing and using health services throughout pregnancy, delivery and postpartum through an accounting methodology. For each incidence of service utilization, three cost categories were used– direct costs related to help seeking, transportation, and other medical and non-medical. Estimates of average per visit costs to case and control households were generated to ensure that the potential difference in number of incidents of help seeking/service utilization among case and control households is controlled for. On the other hand, average total costs were also reported across case and control households to fully understand the financial burden of these costs. In the absence of clinical information, the study could not compare the costs incurred by women who died and who survived the same morbidity to generate the “differential” financial costs of mortality.

In order to assess the impact of these out-of-pocket costs on households, two measures of household economic status were generated. Household consumption expenditure estimates used data collected on expenditures on food and non-food items as well as durable goods (30 food items, 22 non-food consumption items and 13 durable items). The final estimates generated used the food and non-food items due to a high number of missing data in durables expenditures and complications in generating rigorous, reliable estimates. Household asset data as well as key variables of household dwelling characteristics were used to construct a wealth index and wealth groupings. Given the considerably small sample size, the wealth groupings assigned to the sample across three wealth strata are only modestly indicative of their placement.

The study also attempted to capture the extent to which maternal death may trigger disruptions in productivity for an affected household, potentially exacerbating the effects of the financial impact of health and related expenditures. In their study, Ye et al use the income data collected from households to estimate the value of lost wages due to maternal death [3]. Meanwhile in this study, the number of days lost from productive activity and shifts in household division of labor and time use were reported without an attempt to monetize them. This decision was dictated by the near absence of wage labor in the communities the study took place.

Specifically, baseline data were collected on the deceased women’s farming, other economic activity, and household-related work prior to their health deteriorating due to maternal causes. Questions were posed on the time use and task responsibilities of members of the deceased women’s household before and after the maternal death, as well as the changes in these members’ pre- and post-mortality economic activity.

The aim of these questions was to understand how households re-arranged their responsibilities and economic activities to fill the gap created by the maternal death. While typical productivity analyses are in monetary terms, given the study site, no attempt was made to translate these changes into monetary value. This methodology extended typical time use studies by trying to document the shifts not only in time, but also in responsibility, before and after a key catastrophic event.

The qualitative data complemented the quantitative by providing a more nuanced narrative of the productivity disruption described above, what it means for different household members’ workload and daily lives, and the emotional costs of the maternal death.

## Study findings

Table 3 below presents the background characteristics of women who died of maternal causes and those that did not.

**Table 3:** The Characteristics of the Women and their Household^1^

	Cases	Controls	p-value
**Mean age of women**	27.3 (n=59)	26.3 (n=86)	0.414

**Age distribution of women**			

*15-19*	10.2% (n=59)	20.9% (n=86)	0.0719***

*20-24*	27.1% (n=59)	26.7% (n=86)	0.9606

*25-29*	35.6% (n=59)	23.3% (n=86)	0.1155

*30-34*	8.5% (n=59)	12.8% (n=86)	0.4032

*35-39*	16.9% (n=59)	11.6% (n=86)	0.3794

*40+*	1.7% (n=59)	4.7% (n=86)	0.3004

**Mean number of children to women**	2.2 (n=45)	2.1 (n=78)	0.817

**Marital status of women**			

*Married monogamous/cohabiting*	57.6% (n=59)	63.9% (n=86)	0.448

*Not married*	27.1% (n=59)	25.6% (n=86)	0.838

*Married polygamous*	15.3% (n=59)	10.5 (n=86)	0.408

**Educational status of women**			

*Primary*	69.5% (n=59)	70.9% (n=86)	0.854

*Secondary*	23.7% (n=59)	18.6% (n=86)	0.466

*Higher*	3.4% (n=59)	3.5% (n=86)	0.975

*Vocational training*	1.7% (n=59)	3.5% (n=86)	0.494

*None*	1.7% (n=59)	2.3 (n=86)	0.789

*Don’t know*	N/A	1.2% (n=86)	0.32

**Professional category of women**			

*Skilled*	45.8% (n=59)	24.4% (n=86)	0.009*

*Unskilled*	25.4% (n=59)	34.9% (n=86)	0.222

*None*	28.8% (n=59)	40.7% (n=86)	0.139

Household size	5.6 (n=45)	4.8 (n=78)	0.065***

1. (*) significance at 1%; (**) significance at 5%; (***) significance at 10%.

On most characteristics, the women were similar. While generally of similar age, , a significantly larger group of controls were in the 15 to 19 year age category compared to cases, a finding contrary to the evidence in the broader literature on the increased risk of maternal complications and death among adolescent girls. About 60% of women were either in monogamous marriages or cohabiting, a little over 10% were not married and the rest were in polygamous marriages. Education levels were surprisingly high among both groups as more than one quarter of women in either group had secondary schooling or higher. The major difference between cases and controls emerged in regard to profession. Almost twice as many of the sampled women who died in childbirth had been in a skilled profession compared to control women who survived their childbirth experience in the same period. Another area of difference between the women in the case and control groups arose at the household level, with the case households being significantly larger than the control households.

Table 4 presents the distribution of case and control households across these three wealth groups. As can be seen, while there were a slightly higher number of cases in the middle and high wealth groups, the difference between cases and controls overall was not found to be statistically significant.

**Table 4:** Distribution of Cases and Control Households across 3 Wealth Groupings and annual per capita consumption expenditure per grouping

	Case households				Control households			
	**% in wealth group**	**Annual per capita consumption expenditure**			**% in wealth group**	**Annual per capita consumption expenditure**		
				
		Mean	Min	Max		Mean	Min	Max

**Lowest**	26.2% (n=11)	28,058 (n=11)	9,723	53,995	38.4% (n=33)	24,621 (n=32)	8,235	46,215

**Middle**	40.5% (n=17)	36,123 (n=15)	11,098	168,517	31.4% (n=27)	34,730 (n=23)	8,831	91,068

**Highest**	33.3% (n=14)	54,576 (n=14)	23,609	101,869	30.2% (n=26)	45,853 (n=23)	8,295	98,011

Household socio-economic status, as measured in terms of annual per capita consumption expenditure, also pointed at no statistically significant difference among case and control households (see Table 4).

### Complications and care seeking during pregnancy, delivery and postpartum

#### Pregnancy complications and care seeking

A significantly larger number of control women, 88%, reported experiencing at least one complication compared to 75% of women in the case group. The types of complications experienced by the case and control women were quite similar in nature and included headaches, blurred vision, febrile illness, severe abdominal pain, shortness of breath and vaginal bleeding. An exception worth noting is that the five reported cases of HIV were all among women in the case group.

A closer look at care-seeking behavior between cases and controls also revealed some differences. Specifically, the percentage of case and control women with complications who sought help did differ significantly (85% of 44 cases with complications sought help while 66% of the 76 controls did so), so did the number of visits made by cases. During pregnancy average number of visits to a health care provider (institutional or non-institutional) was 2.53 among cases and 1.94 among controls. At the same time, there were no large differences between case and control groups in the type of care sought (Table 5). For both groups, government and mission facilities were the most common places followed by private hospitals and clinics. Significantly more cases had at least one visit with a private provider than controls. Similar to what is reported elsewhere in the literature, very few women used non-institutional services during pregnancy.

**Table 5:** Care seeking among case and control women during pregnancy^1^

	Cases	Controls	p-values
**Total number of women**	59	86	

% who reported complications	74.6%	88.4%	0.042**

% of those with complications who sought care	84.6%	65.8%	0.021**

**Total number of visits for care**	76	97	

**Average number of visits for care^2^**	2.53	1.94	0.043**

**Where sought care (%)^3^**			

*Government/mission health center/clinic*	58%	68%	0.346

*Government/mission hospital*	52%	34%	0.120

*Private clinic*	12%	0%	0.044**

*Private hospital*	6%	6%	0.991

*Pharmacy/duka*	6%	4%	0.685

*Traditional healer*	3%	2%	0.778

*Retired/current practitioner* (*informal*)	0%	2%	0.322

1. (*) significance at 1%; (**) significance at 5%; (***) significance at 10%.

2. Average number of visits for care is calculated for women who sought care only – not the full sample. Of the 33 cases, we know the number of visits for 30 – those 30 made a total of visits and the 50 controls made a total of 97 visits

3. Women might have sought help in more than one type of service

#### Delivery circumstances

A significant number of maternal deaths (14 out of the 59) took place during the last 3 months of pregnancy. One additional woman died during labor, before giving birth. Thus while a total of 45 cases reached labor, 44 among them gave birth. Costs reported during delivery are those incurred by the 45 case households as well as 86 control households

During labor and delivery, the help-seeking behavior of cases and controls showed significant differences. While all controls made visits to a provider during this phase, only 40 out of 45 cases visited a provider during the same time frame. At the same time, with an average of 1.23 visits, cases appear to have gone to significantly more multiple providers, most likely due to referrals to address complications (p-value=0.0642) Cases were also significantly more likely to have delivered at a hospital (p-value=0.066) (Figure 1).

Visits to non-institutional providers were much more common during labor compared to the pregnancy, with about one-third of all visits during labor being made to such providers. While home deliveries were common among cases, the home of the TBA was more commonly reported among controls.

Women who died during or after delivery were less likely to have had a normal delivery and more likely to have gone through a Caesarean section. In fact, almost all women who reported having a normal vaginal delivery (98%), compared to two-thirds of women who suffered a maternal death.

#### Post-delivery complications and care seeking

Of the 39 case women for whom time of death information was available, 14 (36%) died within the first 48 hours of delivery. During this period, about two-third sought care postpartum, a significantly higher rate than reported by controls (Table 6). Among those who sought care, both cases and controls favored government or mission health facilities to others.

**Table 6:** Care seeking among case and control women postdelivery^1^

	Cases	Control	p-values
**Total number of women with delivery information**	44	86	

% who sought care post delivery	61.4%	18.6%	0.000*

**Total number of visits for care after delivery**	45	22	

**Average number of visits for care after delivery^2^**	1.73	1.38	0.269

**Where sought care (%)^3^**			

*Government/mission hospital/health center or clinic*	85.2	93.8	0.365

*Private hospital/clinic*	18.5	6.25	0.220

*Religious leader*	3.7	0	0.327

1. (*) significance at 1%; (**) significance at 5%; (***) significance at 10%.

2. Average number of visits for care is calculated for women who sought care only – not the full sample. Of the 33 cases, we know the number of visits for 30 – those 30 made a total of visits and the 50 controls made a total of 97 visits

3. Women might have sought help in more than one type of service

### Financial costs and impact on households of help seeking during pregnancy, labor and postpartum

Similar to what emerged in other recent studies, the health service utilization costs associated with maternal deaths were significantly higher, due to more frequent service utilization as well as due to the higher cost of each visit suggesting more involved treatments and interventions were sought with these women (Table 7).

**Table 7:** Average per visit cost of health care utilization during pregnancy, delivery and post-partum (in KES)^1,2^

	Pregnancy			Delivery and Postpartum		
	
	Cases	Controls	p-value	Cases	Controls	p-value
*Fees*	1775,2 (n=28)	222.2 (n=50)	0.006*	5324,1 (n=34)	1396,6 (n=85)	0.076***

*Transport*	399 (n=29)	87,9 (n=50)	0.104	246,2 (n=36)	145,6 (n=86)	0.024**

*Purchases^3^*	723,2 (n=27)	150,4 (n=50)	0.025**	329,3 (n=34)	166,5 (n=84)	0.069***

*Total*	993,2 (n=29)	153,5 (n=50)	0.007*	1921,2 (n=36)	563,2 (n=86)	0.059***

1. Includes both institutional and non/institutional heath care.

2. (*) significance at 1%; (**) significance at 5%; (***) significance at 10%.

3. Purchases include other medical and non-medical expenses such as drugs bought from outside of the medical facility, bedding, food and hotel stay for women and their company

The already high costs incurred by cases during pregnancy were further increased during delivery and postpartum mainly a result of higher facility-based fees and expenses.

Adding to these facility-based expenses, the variation with respect to the average household spending on transport costs to and from the health facility during pregnancy was quite sizeable between case and control households**.** Upon closer analysis, the data also revealed that cases had a greater number of individuals accompanying them on visits which, along with modes of transport, explain the difference.

The financial impacts of costs on households are more easily understood in light of their annual per capita consumption expenditure. A pattern consistent across both cases and controls is the steady increase in spending with households in the higher wealth category on average spending more (Figure 2). This is not surprising, as one may expect an increasing willingness and ability to pay from wealthier households.

More noteworthy is that the poorest households among the cases paid more for health care costs associated with the pregnancy than the wealthiest households among the controls, who survived pregnancies.

As can be seen in Table 8, this is reinforced in terms of the share that these costs constitute in households’ per capita consumption expenditure. For cases across the three wealth groupings, these costs approach about one-third of their per capita consumption expenditure. For controls, at the most, the share remains at 12%.

**Table 8:** Average total costs as % of household consumption expenditure, by wealth groupings (in KES)

	Pregnancy		Delivery and postpartum		Total	
	**Cases**	**Controls**	**Cases**	**Controls**	**Cases**	**Controls**

**Lowest**	12%	2%	22%	3%	34%	5%

**Middle**	13%	3%	19%	4%	32%	7%

**Highest**	23%	3%	10%	9%	33%	12%

**Table 9:** Funeral costs, total and as percentage of per capita consumption expenditure

	Total funeral costs (KES)	As % of annual per capita household consumption expenditure
**Lowest (n=6)**	28,554	102

**Middle (n=16)**	53,282	148

**Highest (n=14)**	66, 974	123

The burden of the health care access and utilization on household budgets, particularly for case households, is apparent. While both case and control households used their saving as the primary source for financing the costs, about 44% of case households had to seek financing outside of the household, versus 21% of controls. Common sources of financial support for case households included community fundraising and welfare groups. Only 25 study households, among them 4 cases, reported having some type of social insurance or waiver and 17 used it towards offsetting some of the costs incurred.

### Funeral costs

Studies from multiple countries in Africa on the costs of adult mortality from causes such as AIDS, TB or malaria have found that funeral costs can be substantial [25-28], at least partly due to cultural practices whereby the family is expected to provide food for close relatives, in-laws and guests who attend the funeral. Studies have also found that households often sell assets or borrow from family and friends to pay for funeral and other expenses related to the death of an adult household member, with the risk of going into further poverty as a consequence [18,26].

The analysis of funeral costs collected from 56 case households reveals a similar pattern. Funeral costs added up to as high as KES 182,500 with households in the highest wealth group spending KES 66,974 on average. Many households had to seek financial support including from family members (87%) and the community (65%). Also, 27% of households reported selling assets, and close to 15% reported seeking assistance from a moneylender to finance funeral costs.

### The impacts of maternal mortality on household economic activity

In the case households, the financial shock stemming from health care and related expenses are coupled with the economic shock that is due to the loss of a productive member of the household, and the ensuing reallocation of time and labor for household work and economic activity.

In most of the case households, the women who died had carried out a significant portion of household tasks, and were involved in farming and other income generating activities to supplement household income.

Women’s household work which included the traditional tasks of fetching water and firewood, childcare and other care work amounted to about 61 hours of their week. As can be seen in Figure 3, husbands took on some of these tasks at the expense of given up other work. As one husband noted: *“…[I] am used to going to the farm early*, *but when she was gone it was a must that I make sure that those children have had something to drink…When I come back from the farm*, *I need to wash the clothes*, *I need to wash them [children]*, *I also need to find them food; all this from? How many people? Me*, *just one person…[with the grandmother’s help].”* (Husband of deceased).

Yet, more commonly it was the other female members of the household who were pulled into meeting the needs of the household. Mothers and the mothers-in-law significantly increased their effort, particularly around care tasks that sustain the family, such as childcare, cooking and laundry.

Alongside their highly significant role in managing the household, the deceased women were economically active, particularly in agriculture. Of the 26 women who were involved in farming, 12 had their own farm, 13 worked in their family farm and 1 had a leased farm. Time use data were available for 22 out of 26 farmer women which revealed that on average 868 minutes (or 14.5 hours) a week on farming activities working on average 7.5 months a year. All 26 women were involved in planting, weeding and harvesting, and 20% also contributed to ploughing and land preparation. About 40% of these women also tended livestock.

Many respondents noted how the deceased was a key part of the farming labor of the household, without whom they would be forced to allow land to lay fallow and/or cultivate fewer crops. Some noted losing crops. Also, as with household tasks, the disruption chain rippled through the household, such that surviving household members could not allocate to farming the time they used to when the deceased was alive. This theme that echoes through the transcripts, is well articulated by one family member who noted:

“Since *this girl is not there*, *I have to come back and make them [household members? Children?] tea*, *or I even make them porridge*, *after that is when I go back to the farm. When it reaches one o clock*, *I come back and see that they have some ‘ugali’ [a staple food made of maize flour cooked in boiling water until stiff] and vegetables*, *I leave when they have already eaten then I go back to the farm*.” (Mother-in-law of deceased).

Others also noted that, for a husband in particular, “*He [the deceased’s spouse] has reduced his working on the farm he does not work on the farm like before when they were together with the deceased*,” (Mother-in-law of deceased).

In most of the households interviewed (74%), there was only one other economically active household member. Among the households who suffered a maternal loss, 43 had members other than the deceased woman who were economically active prior to the loss. Most typically, these were the spouses of women (33) but also their parents (15) and mothers-in-law (4). Overall, on average 16 days were taken off by these members during maternal illness and another 26 days to meet funeral obligations. Over two-thirds of these household members had to take time off during the illness for an average of 22 days. All of them also took time off during the funeral for about 28 days. In aggregate terms, this translates to the only economically active household member taking off about 2 months off from their work to support women during their illness and to tend to their funeral.

A majority of case households (38) also saw a significant impact on their work in the longer–term, as they had reduced their time on economic activity. For example, in some households, mothers-in-law had to give up their wage labor – and the income it brought in – to take care of the children left behind by the deceased woman. In others, husbands took on extra responsibilities that they were unused to. Only in five cases – all among spouses – was there an increase in time allocated on productive activity.

## Discussion

Maternal death is a highly traumatic event that affects the households at many levels. This paper presents evidence on one of these many aspects of maternal morbidity and death; the extent of the financial burden faced by household members. What emerges clearly is that throughout various stages of pregnancy and childbirth, even if the self-or relative-reported complications among cases and controls did not reveal significant differences, women who eventually succumbed to complications had more frequent visits to health care providers, and incurred higher total costs at each visit.

At the end, their households spent about 30% of their annual per capita consumption expenditure to cover these costs. The burden of these health care related costs were further exacerbated significantly by the costs incurred by households in funeral expenses. What is more, very few households were supported by an insurance scheme to help meet these costs, particularly health care costs, and were forced to adopt strategies such as dissaving, borrowing money and liquidating assets. There seemed to be more community support to families in meeting funeral costs.

The financial shock experienced by households is likely to be particularly catastrophic in light of the immediate productivity impacts they face. Despite some data gaps that impose limitations to the analysis, what emerge clearly are the multiple productive and reproductive roles the deceased women used to have and the immediate disruption their death had caused. Among the surviving household members, the division of labor and time use changed as remaining members took on household work. At the height of the health crisis and the eventual death, economically active household members took substantial amount of time off from productive work making the mitigation of the financial costs even harder. For many of these household members, the change in their level of economic engagement was long lasting as many reported reduced time on their economic activity, which often takes place at the household level and have direct bearing on household subsistence.

Maternal mortality is increasingly concentrating in developing countries and within these countries, among the poorer populations [29-31]. Both the immediate and long-term economic shocks of maternal death only add to the vulnerability of poor households who already face financial uncertainty and seldom have the institutionalized social protection.

Kenya is undergoing a legal reform process that would pave the way for free maternity services in all public facilities. Such a policy if implemented effectively with considerations to the quality of care can lift a major economic burden experienced by a very large number of household who seek maternal health services which can be catastrophic in complicated cases that result in maternal death. Also while there were various insurance and waiver schemes in place in the study area, many households were relying on more informal social support. It would be important to understand the underlying reasons for this pattern and find ways to incentivize participation in health insurance schemes that can safeguard households from facing catastrophic health expenditures, such as those related to maternal morbidity and mortality. Funeral insurance, which had gained traction during the HIV epidemic, can be part of such schemes.

## Competing interests

The authors declare that they have no competing interests.

## Peer review

Reviewer reports for this article can be found in Additional file 1.

## Supplementary Material

Additional file 1 Click here for file

## Declarations

This article has been published as part of *Reproductive Health* Volume 12 Supplement 1, 2015: True costs of maternal death. The full contents of the supplement are available online at http://www.reproductive-health-journal.com/supplements/12/S1. Publication charges for this supplement were funded by Family Care International and the FXB Center for Health and Human Rights.
