# Supplementary material for: The economic burden of maternal mortality on households: evidence from three sub-counties in rural western Kenya
Source: Reprod Health. 2015 May 6;12(Suppl 1):S3. doi: 10.1186/1742-4755-12-S1-S3 (PMC4423575; doi:10.1186/1742-4755-12-S1-S3)
Supplement: Additional file 1 [file 1742-4755-12-S1-S3-S1.pdf]

Referee's comments to the authors– this sheet WILL be seen by the author(s) and published with the article

Title	The Economic Burden of Maternal Mortality on Households: Evidence from Three sub-Counties in Rural Western Kenya
Author(s)	Aslihan Kes, Sheila Ogwang, Rohini Prabha Pande, Zayid Douglas, Robinson Karuga, Frank O Odhiambo, Kayla Laserson, and Kathleen Schaffer
Referee's name	George Kosimbei

When assessing the work, please consider the following points, where applicable:

1. **Is the question posed by the authors new and well defined?**
Yes, the question is well defined though it can be made clearer by pinpointing that the paper is about expenditures rather than costs. Costs are a different thing all together.

2. **Are the methods appropriate and well described, and are sufficient details provided to replicate the work?**
The methods are appropriate though we need to know the details of the questions posed on this questionnaire so as to capture the expenditures accurately. When the households are reporting the expenditures what steps did the researchers take to ensure that they were realistic? Did they want to see some evidence on the same?

How did the study address the problem of confounding in articulating the IMPACT?

3. **Are the data sound and well controlled?**

The data generation process is sound. The only issue is the verification of the expenditure data reported by the household. The details of the verification process need to be clear.

4. **Does the manuscript adhere to the relevant standards for reporting and data deposition?**
The manuscript adheres to the standards of reporting only that most of the figures are missing

5. **Are the discussion and conclusions well balanced and adequately supported by the data?**
The discussion section needs to be beefed up especially on the issue of catastrophic health expenditures
The poverty dimension needs to be very clear.

6. **Do the title and abstract accurately convey what has been found?**
The title should be on expenditure
7. **Is the writing acceptable?**
The writing is acceptable though grammar should be corrected

Please make your report as constructive and detailed as possible in your comments so that authors have the opportunity to overcome any serious deficiencies that you find and please also divide your comments into the following categories:

- Major Compulsory Revisions (which the author must respond to before a decision on publication can be reached)
- Minor Essential Revisions (such as missing labels on figures, or the wrong use of a term, which the author can be trusted to correct)
- Discretionary Revisions (which are recommendations for improvement but which the author can choose to ignore)

Where possible please supply references to substantiate your comments.

When referring to the manuscript please provide specific page and paragraph citations where appropriate.

General comments:
The article is important especially now that Kenya is putting a lot of effort in maternal health. There needs to be clarity on expenditure rather than costs

Major compulsory revisions:

The methods are appropriate though we need to know the details of the questions posed on this questionnaire so as to capture the expenditures accurately.
When the households are reporting the expenditures what steps did the researchers take to ensure that they were realistic?
Did they want to see some evidence on the same?

How did the study address the problem of confounding in articulating the IMPACT?

The data generation process is sound. The only issue is the verification of the expenditure data reported by the household. The details of the verification process need to be clear.

(continue on the next sheet)

Continued:
The discussion need to be beefed up especially on the issue of catastrophic health expenditures
The poverty dimension needs to be very clear.

Referee's comments to the authors– this sheet WILL be seen by the author(s) and published with the article

Title	The Economic Burden of Maternal Mortality on Households: Evidence from Three sub-Counties in Rural Western Kenya
Author(s)	Aslihan Kes, Sheila Ogwang, Rohini Prabha Pande, Zayid Douglas, Robinson Karuga, Frank O Odhiambo, Kayla Laserson, and Kathleen Schaffer
Referee's name	Scott Grosse

When assessing the work, please consider the following points, where applicable:

1. Is the question posed by the authors new and well defined?
2. Are the methods appropriate and well described, and are sufficient details provided to replicate the work?
3. Are the data sound and well controlled?
4. Does the manuscript adhere to the relevant standards for reporting and data deposition?
5. Are the discussion and conclusions well balanced and adequately supported by the data?
6. Do the title and abstract accurately convey what has been found?
7. Is the writing acceptable?

Please make your report as constructive and detailed as possible in your comments so that authors have the opportunity to overcome any serious deficiencies that you find and please also divide your comments into the following categories:

- Major Compulsory Revisions (which the author must respond to before a decision on publication can be reached)
- Minor Essential Revisions (such as missing labels on figures, or the wrong use of a term, which the author can be trusted to correct)
- Discretionary Revisions (which are recommendations for improvement but which the author can choose to ignore)

Where possible please supply references to substantiate your comments.

When referring to the manuscript please provide specific page and paragraph citations where appropriate.

General comments:

Major compulsory revisions:
The Methods section should acknowledge that the study used basically the same approach as used in the Chinese study by Ye et al., with one important exception. The present study chose not to express lost productivity in monetary terms. Rather than representing an "innovation" as claimed on page 7, the inclusion of estimates of lost days of work represents a valuable replication in a different setting.

Also, the study shares both the strengths and limitations of the Chinese study.

Minor essential revisions:

(continue on the next sheet)

Continued:
In particular, there is a major limitation that was not acknowledged in either paper. Neither study made any attempt to distinguish healthcare costs attributable to maternal death from those of the maternal morbidity that led to death. As an economist, I disagree that the authors have calculated the costs associated with maternal death. Rather, they have calculated the average difference of healthcare utilization and costs between two groups of women: those who gave birth and survived, most of whom were healthy, and those who gave birth and died due to illness or injury. An accurate estimation of the cost attributable to maternal death per se would have required the authors to compare costs for pregnant women who had the same health issues, e.g., postpartum hemorrhage, and either survived or died. I suspect that most, if not all, of the difference in mean cost of healthcare use is attributable to maternal morbidity rather than death.

I am not suggesting that the analysis should be altered, but the interpretation of the results must be changed to indicate that the estimates of healthcare costs probably reflect the impact of the underlying maternal morbidity that led to death rather than the effect of maternal death. This difference in interpretation has important policy implications. Averting maternal death due to better quality maternity care would likely not reduce the burden of healthcare costs because those costs reflect treatment of maternal morbidity, not death. Therefore, it is more reasonable in my opinion, to exclude the healthcare costs from an estimation of the economic impact of maternal death. On the other hand, if the authors reframe their analysis as an estimate of the impact of severe maternal morbidity that results in death in comparison to costs associated with uncomplicated pregnancy and childbirth, the reported estimates would appear to be correct.

Supplement Editor comments

There are too many footnotes. The style of the journal is to have footnotes only as an exception. Please try to avoid them.
Please follow the Instructions for authors regarding the tables and references. Tables should be at the end of the manuscript. One page per table.
Regarding references the journal style is to numbered references following the order that they appear in the text. Also please follow the Vancouver style for references.
